# Engineered endolysin of *Klebsiella pneumoniae* phage is a potent and broad-spectrum bactericidal agent against “ESKAPEE” pathogens

**DOI:** 10.3389/fmicb.2024.1397830

**Published:** 2024-05-09

**Authors:** Wei Chen, Li-Mei Han, Xiu-Zhen Chen, Peng-Cheng Yi, Hui Li, Yun-Yao Ren, Jing-Han Gao, Cai-Yun Zhang, Jing Huang, Wei-Xiao Wang, Zhi-Liang Hu, Chun-Mei Hu

**Affiliations:** ^1^Department of Tuberculosis, The Second Hospital of Nanjing, Affiliated to Nanjing University of Chinese Medicine, Nanjing, China; ^2^Clinical Research Center, The Second Hospital of Nanjing, Affiliated to Nanjing University of Chinese Medicine, Nanjing, China; ^3^Department of Infectious Diseases, The Second Hospital of Nanjing, Affiliated to Nanjing University of Chinese Medicine, Nanjing, China; ^4^Department of Laboratory Medicine, The Second Affiliated Hospital of Nanchang University, Nanchang, China; ^5^Department of Clinical Laboratory, The Second Hospital of Nanjing, Affiliated to Nanjing University of Chinese Medicine, Nanjing, China; ^6^Center for Global Health, School of Public Health, Nanjing Medical University, Nanjing, China; ^7^Innovation Center for Infectious Diseases of Jiangsu Province, Nanjing, China

**Keywords:** ESKAPEE, polymicrobial infection, endolysin, engineering, ApoE23, COG133, coinfection bacteremia

## Abstract

The rise of antimicrobial resistance in ESKAPEE pathogens poses significant clinical challenges, especially in polymicrobial infections. Bacteriophage-derived endolysins offer promise in combating this crisis, but face practical hurdles. Our study focuses on engineering endolysins from a *Klebsiella pneumoniae* phage, fusing them with ApoE23 and COG133 peptides. We assessed the resulting chimeric proteins’ bactericidal activity against ESKAPEE pathogens *in vitro*. ApoE23-Kp84B (CHU-1) reduced over 3 log units of CFU for *A. baumannii*, *E. faecalis*, *K. pneumoniae* within 1 h, while COG133-Kp84B (CHU-2) showed significant efficacy against *S. aureus*. COG133-L1-Kp84B, with a GS linker insertion in CHU-2, exhibited outstanding bactericidal activity against *E. cloacae* and *P. aeruginosa*. Scanning electron microscopy revealed alterations in bacterial morphology after treatment with engineered endolysins. Notably, CHU-1 demonstrated promising anti-biofilm and anti-persister cell activity against *A. baumannii* and *E. faecalis* but had limited efficacy in a bacteremia mouse model of their coinfection. Our findings advance the field of endolysin engineering, facilitating the customization of these proteins to target specific bacterial pathogens. This approach holds promise for the development of personalized therapies tailored to combat ESKAPEE infections effectively.

## 1 Introduction

“ESKAPEE” refers to a group of notorious drug-resistant bacterial pathogens comprising *Enterococcus faecium, Staphylococcus aureus, Klebsiella pneumonia, Acinetobacter baumannii, Pseudomonas aeruginosa, Enterobacter species, and Escherichia coli* (Rice, 2008; Rajput et al., 2021; Neidhöfer et al., 2023). These pathogens share a common trait: their ability to evade the effects of conventional antibiotics through various mechanisms, including overexpressed efflux pumps, increased biofilm production, reduced cell wall permeability, and horizontal gene transfer (Santajit and Indrawattana, 2016). The emergence of antimicrobial resistance among these pathogens has significantly restricted treatment options for serious infections, resulting in a substantial increase in disease burden attributed to treatment failures (de Oliveira et al., 2020). Notably, these pathogens are predominantly responsible for the deadliest hospital-acquired infections (HAIs), resulting in significant mortality annually (Ayobami et al., 2022; Abban et al., 2023). A systematic review and meta-analysis showed that the weighted prevalence of HAIs was 3.13% in mainland China from 2006 to 2016 (Wang et al., 2018). Furthermore, polymicrobial infections involving ESKAPEE bacteria exacerbate the complexity of clinical scenarios. For instance, *Acinetobacter baumannii* complex (ABC) infections often manifest as polymicrobial infections (Karakonstantis et al., 2022). Of particular concern is coinfection with *Enterococci*, which complicates pathogenesis, worsens prognoses, and undermines the efficacy of antimicrobial agents by fostering the colonization, proliferation, and persistence of various pathogenic bacteria (Xu et al., 2024). Therefore, addressing the therapeutic challenges posed by polymicrobial infections of ESKAPEE is of paramount importance and combinatorial therapies or innovative antimicrobial agents are urgently needed.

Endolysins are peptidoglycan hydrolases expressed by phages to release offspring viral particles at the end of the phage replication cycle, which have emerged as promising antimicrobial agents (Rahman et al., 2021). Most endolysins from phages infecting Gram-positive bacteria have a modular structure, comprising one or two N-terminal enzymatically active domains (EADs) linked by a flexible linker to a C-terminal cell wall-binding domain (CBD). In contrast, endolysins from phages infecting Gram-negative hosts typically consist of a simple globular EAD module without a CBD. EADs are classified into three categories based on their mode of action and enzymatic specificities: glycosidases, amidases, and endopeptidases (Abdelrahman et al., 2021). Endolysins offer several advantages, including high barrier of resistance development, rapid bacterial cell lysis, synergy with other antibiotics, and effectiveness in biofilms eradication (Rahman et al., 2021). Over the past decade, there has been a resurgence of interest in endolysin therapy, resulting in numerous successful applications (Khan et al., 2023). For example, endolysin PlyBa, Ply12, and Ply21 are applied to control *Bacillus cereus*, as well as many other strains of *B. thuringiensis* in food industry (Loessner et al., 1997). Staphefekt SA.100, a topical chimeric endolysin developed by a Dutch biotech company Micreos, has been available for human use in Europe since 2017 (Herpers et al., 2014). However, practical challenges persist in the use of endolysins. Despite the abundance of endolysins in nature, discovering novel and potent variants necessitates several steps, including phage isolation, propagation, endolysin cloning, and purification. Some natural endolysins exhibit poor expression levels and solubility (Lee et al., 2023). Moreover, while these “enzybiotics” effectively eliminate Gram-positive bacteria through specific cell wall hydrolysis (Liu et al., 2023), their application is hindered by the protective outer membrane of Gram-negative bacteria (Rahman et al., 2021). Genetic modification of current endolysins offer a solution to tailor their properties and activity against specific bacterial targets. “Artilysins,” based on endolysin engineering, represent promising alternatives to combat multidrug-resistant bacterial infections (Briers et al., 2014). Studies have shown that fusion with membrane-penetrating sequences containing positively charged amino acids enhances bactericidal activity (Carratalá et al., 2023). However, the precise determinants of lytic host range and bactericidal activity of engineered endolysins remain unclear. Consequently, endolysin engineering continues to rely on empirical approaches.

Apolipoprotein E (ApoE) is a protein that associates with lipid particles and plays a key role in lipoprotein-mediated lipid transport between organs (Litvan and Saunders, 1998). Mimetic peptides derived from ApoE have shown promising properties across various disease contexts, suggesting their potential as therapeutic candidates (Vitek et al., 2012; Wolska et al., 2021; Nankar et al., 2022). Notably, some of these peptides exhibit antimicrobial activity through membrane disruption, as well as immunomodulatory effects (Wang et al., 2013, 2022, 2023; Pane et al., 2016). For example, ApoE23, consists of 23 amino acids (LRKLRKRLVRLASHLRKLRKRLL), derived from a combination of residues 141–148 and 135–149 of ApoE (Wang et al., 2023). Similarly, COG133 is a 17-amino acid peptide (LRVRLASHLRKLRKRLL), corresponding to residues 133–149 of ApoE protein (Zhang et al., 2022). Both ApoE23 and COG133 are cationic antimicrobial peptides with similar sequences and compositions. Investigating the antimicrobial activity and spectrum could yield valuable insights into endolysin engineering, when the peptides fuse with endolysins.

In this study, we isolated a lytic phage and engineered its endolysin by fusing it with ApoE23 and COG133, respectively. Then we evaluated the bactericidal activities of the resulting chimeric proteins against ESKAPEE pathogens *in vitro* and assessed their protective efficacy against coinfection with *A. baumannii* and *E. faecalis in vivo*. Our findings contribute to the advancement of endolysin engineering, enabling the customization of their properties and activity against specific bacterial targets. This approach holds promise for the development of personalized therapies tailored to combat ESKAPEE infections.

## 2 Materials and methods

### 2.1 Bacterial strains, phages, media, and growth conditions

The bacterial strains and phages used in this study are listed in Table 1. Clinically isolated strains were sourced from the Sample Library of the Second Hospital of Nanjing. Initial bacterial identification was conducted using Vitek 2.0 and matrix-associated laser desorption ionization-time of flight mass spectrometry (MALDI-TOF MS; BioMerieux, Craponne, France). Details of their antibiotic resistances are documented in Supplementary Table S1. All bacteria, with the exception of *E. faecalis*, were cultured in LB agar or broth at 37°C with agitation. *Enterococcus faecalis* was cultivated in brain heart infusion (BHI) medium. When required, 50 μg/mL carbenicillin was supplemented to maintain plasmids in *E. coli*.

**Table 1:** Bacteria, phages, and plasmids used in this study.

	Characteristics	References or sources
**Bacteria**		
*Escherichia coli* Stellar	Routinely used host for plasmid construction	Takara Bio
*Escherichia coli* BL21 (DE3)	Host for protein expression	Laboratory strain
*Acinetobacter baumannii* YQ4	Pandrug-resistant clinically isolated strain	Wang et al. (2022)
*Enterobacter cloacae* 7-26	Carbapenem-resistant clinically isolated strain	This study
*Enterococcus faecalis* 10-17	Vancomycin-resistant clinically isolated strain	This study
*Klebsiella pneumoniae* 84	Pandrug-resistant clinically isolated strain	This study
*Pseudomonas aeruginosa* 1–22	Carbapenem-resistant clinically isolated strain	This study
*Staphylococcus aureus* 14-32	Methicillin-resistant clinically isolated strain	This study
**Phages**		
ФKp84B	Lytic phage of *K. pneumoniae* 84	This study
ФAb4B	Lytic phage of *A. baumannii* YQ4	Laboratory storage
ФEF10-17	Lytic phage of *E. faecalis* 10–17	Laboratory storage
**Plasmids**		
pET15b	Plasmid for protein expression, T7 promoter, CBC^r^	Laboratory vector
pET15b-LysKp84B	Expressing LysKp84B based on pET15b, CBC^r^	This study
pET15b-CHU-1	Expressing CHU-1 based on pET15b, CBC^r^	This study
pET15b-CHU2	Expressing CHU-2 based on pET15b, CBC^r^	This study
pET15b-COG133-L1-Kp84B	Expressing COG133-L1-Kp84B, CBC^r^	This study
pET15b-COG133-L2-Kp84B	Expressing COG133-L2-Kp84B, CBC^r^	This study
pET15b-COG133-L3-Kp84B	Expressing COG133-L2-Kp84B, CBC^r^	This study
pET15b-COG133-L4-Kp84B	Expressing COG133-L3-Kp84B, CBC^r^	This study

The lytic phages ФAb4B and ФEF10-17 were isolated previously from untreated wastewater of the Second Hospital of Nanjing, using *A. baumannii* YQ4 and *E. faecalis* 10-17 as hosts, respectively.

### 2.2 Phage isolation and identification

Phage ФKp84B was isolated from untreated wastewater collected at the Second Hospital of Nanjing using the standard double-layered plating technique (Peters et al., 2022). The wastewater underwent filtration through a 0.22 μm membrane, followed by overnight incubation after mixing with the bacterial culture of *Klebsiella pneumoniae* 84 (Kp 84). The resulting supernatant was harvested via centrifugation, combined with the bacterial culture, and poured onto LB double-layer agar plates. Clear plaques were observed after overnight incubation at 37°C, and selected plaques were picked into 1 × PBS buffer. This entire phage isolation and purification procedure was repeated three times.

The phage titer was determined through a double agar overlay plaque assay following established protocols (Kropinski et al., 2009). After purification via CsCl gradient ultracentrifugation and dialysis, the morphology of ФKp84B was examined using transmission electron microscopy (TEM) at the Center of Electron Microscopy, Agriculture University of Nanjing. Specifically, the phage lysate was filtered through a 0.22 μm filter membrane, deposited onto copper grids, negatively stained with 2% uranyl acetate, and air-dried using filter paper. Phages were visualized using a Hitachi HT7700 TEM.

Genomic DNA of phage ФKp84B was extracted following the manufacturer’s instructions provided with the Isolation Kit of Virus Genomic DNA (Solarbio, Cat. No. D2410). Genome sequencing was conducted using the Illumina NovaSeq 6000 platform (Magigene, China), and *de novo* assembly of clean data was performed using Megahit software (v1.1.2). Genomic DNA annotation was carried out using Prokka, and the function of each Open Reading Frame (ORF) was manually identified using BLASTp.

### 2.3 Plasmid construction

The plasmids used in this study are listed in Table 1. All plasmid construction was performed using the In-Fusion cloning method following the manufacturer’s manual. The primers used in this study are listed in Supplementary Table S2. For expression LysKp84B, the open reading fragment of LysKp84B was amplified using the genomic DNA of ФKp84B as template, and cloned into pET15b cut by NdeI, generating pET15b-LysKp84B. To insert ApoE23 or COG133 at the N-terminus of LysKp84B, pET15b-LysKp84B was linearized by reverse PCR and fused with oligonucleotide fragment ApoE23 or COG133, generating pET15b-ApoE23-LysKp84B (CHU-1) and pET15b-COG133-LysKp84B (CHU2). To insert GS linker to pET15b-CHU2, GS linker fragment was prepared by annealing and fused with linearized pET15b-COG133-LysKp84B. Surprisingly, even though we planned to insert L4, we got insertion of L1, L2 and L3 by accident, which might be due to recombination within the plasmid.

### 2.4 Protein expression and purification

The derivative plasmids of pET15b were transformed into chemically competent cells of *E. coli* BL21 (DE3). These transformants were cultured until reaching the log phase at 37°C, following which protein expression was induced by the addition of 1 mM IPTG at 16°C overnight. Bacterial cells were then harvested and suspended in lysis buffer (25 mM PBS, 500 mM NaCl, and 40 mM imidazole, pH 7.4), and subsequently disrupted by sonication using an Intelligent Ultrasonic Processor (XO-650D) with the following conditions: 45% amplitude, 5 s on and 5 s off, for a total duration of 30 min. After centrifugation at 12,000 rpm for 60 min, the supernatant containing the target proteins was collected and passed through a Ni-NTA superflow column (Cytiva) for protein purification, as per the manufacturer’s instructions. Buffer exchange was carried out using an ultrafiltration column (Millipore) to transfer the proteins into a storage buffer (20 mM Tris–HCl and 50 mM NaCl, pH 7.4). The purity of the purified protein was assessed using 12% SDS-PAGE, while the protein concentration was determined using a bicinchoninic acid protein assay kit (Beyotime). For use *in vivo*, endotoxin in the protein solution was removed by Triton X-114 following the optimized protocol (Teodorowicz et al., 2017). The residual level of endotoxin was measured by Gel Clot Endotoxin Assay Kit (Bioendo, Xiamen). Finally, the purified proteins were aliquoted and stored at −80°C until further use.

### 2.5 Zymogram analysis

The zymographic assay was performed following a previously established protocol (Fukushima and Sekiguchi, 2016). Briefly, 50 μg of LysKp84B with or with 10 μ M Zn^2+^ was boiled for 5 min in sample buffer (62.5 mM Tris–HCl, pH 6.8, 2% SDS, 20% glycerol, 5% β-mercaptoethanol, 0.1% bromophenol blue) and then loaded onto 12% polyacrylamide gels containing 5 mg of peptidoglycan (Weikeqi, Sichuan) and 0.001% SDS. As a negative control, 5 μg of Bovine serum albumin (BSA) was used. The gels were run at 80 V for 30 min followed by 150 V for 60 min. Subsequently, the gel was washed with water for 15 min and then incubated at 37°C for 16 h in 1% Triton X-100 and 25 mM Tris–HCl, pH 7.8. After another wash with water, the gel was stained for 2 h with 0.5% methylene blue in 0.01% KOH and then destained with water. Peptidoglycan hydrolase activity appeared as a clear zone against the blue background of stained peptidoglycan. Gels not containing peptidoglycan were stained with Coomassie Brilliant Blue.

### 2.6 Determination of the lytic activity of engineering endolysins

To measure the bactericidal activity of endolysin, the bacteria were cultured to the log phase, washed once, and resuspended in 20 mM Tris–HCl buffer (pH 7.4). The optical density at 600 nm (OD_600_) of the bacterial culture was adjusted to 0.5, corresponding to approximately 1 × 10^8^ CFU/mL, followed by dispensing into 1.5 mL Eppendorf tubes, with 100 μL in each. Then, 10 μM protein were added in a 150 μL reaction system. To address the effect of Zn^2+^, 10 μM ZnSO_4_ was supplemented. Two replication tubes were set up for each bacterium and protein. 10 μM ApoE23 or COG133 (GL Biochem, Shanghai) was used as a positive control. 10 μM zinc sulfate and no drug buffer were negative controls. After incubation at 37°C for 1 h, the reaction mixture was gradiently diluted and dropped onto agar plates for enumeration of viable cells. The assay for the lytic activity was performed in triplicate.

### 2.7 Confocal laser scanning microscopy

The assessment of CHU-1-induced damage to both *A. baumannii* and *E. faecalis* was conducted following a previously established protocol (Farkas et al., 2017), employing the Live/Dead BacLight bacterial viability kit (Invitrogen L7012). The log-phase culture of Ab YQ4 or Ef 10-17 was harvested, washed once and resuspended in 20 mM Tris–HCl (pH 7.4). The suspension was then incubated for 1 h at 37°C with or without 10 μM CHU-1 and ZnSO4. Subsequently, the cells were stained with 7.5 μM SYTO-9 and 30 μM propidium iodide (PI) in the dark for 15 min. 5 μL of the culture were then spotted onto a clean slide coated with a thin layer of 1% agarose. Fluorescence imaging was performed using the ZEISS LSM900 confocal laser scanning microscope. SYTO-9 fluorescence was detected with excitation at 483 nm and emission at 503 nm, while PI fluorescence was observed with excitation at 493 nm and emission at 636 nm.

### 2.8 Scanning electron microscopy

The effect of engineered endolysins on bacterial morphology was evaluated using scanning electron microscopy (SEM). Bacterial strains were cultured to the log phase. After two washes with 20 mM Tris–HCl (pH 7.4), the bacterial pellets were resuspended and incubated with or without engineered endolysin at 37°C for 1 h. In detail, Ef 10-17, Ab YQ4 and Kp 84 were treated by CHU-1. Sa 14-32 was treated by CHU-2. Pa 1-22 and Ecl 7-6 were treated by COG133-L1-Kp84B. Following incubation, the suspension was centrifuged at 12,000 rpm for 1 min, and the residual pellets were fixed with 2.5% buffered glutaraldehyde at 4°C for 2 h, then post-fixed in 1% buffered osmium tetroxide for 2 h. Subsequently, the pellets were dehydrated in a graded series of ethanol, frozen in liquid nitrogen-cooled tert-butyl alcohol, and vacuum-dried overnight. Finally, the bacterial pellets were mounted onto aluminum stubs, vacuum sputter-coated with gold, and the membrane morphology was analyzed using a Hitachi S-3000 N SEM (Japan).

### 2.9 Biofilm eradication assay

The static biofilm was prepared as previously described (Merritt et al., 2005; Fursov et al., 2020). The log-phase cultures of AbYQ4 and Ef 10-17 were separately transferred to the 96-well polyvinyl chloride (PVC) plate and incubated for 24 h at 37°C to allow biofilm formation. To promote biofilm formation, 1% glucose was supplemented into LB or BHI broth. After incubation, unattached cells were removed, and the wells were washed once with 20 mM Tris–HCl buffer (pH 7.4). Subsequently, the wells were treated with 10 μM CHU-1 for another 24 h at 37°C. Positive controls, including 0.5 M EDTA, 10 μM antimicrobial peptide, and 10 μM LysKp84B, were separately used. Furthermore, phages ФAb4B and ФEF10-17 were employed as positive controls for *A. baumannii* and *E. faecalis*, respectively, with a multiplicity of infection (MOI) of 1. Meanwhile, 10 μM LysKp84B and 10 μM antimicrobial peptide were used simultaneously. Zinc sulfate (10 μM) was always added along with endolysin. For each sample, 8 replication wells were set up. After treatment, non-adherent cells were removed by washing the plate with sterile water three times. The wells were then stained with 1% w/v crystal violet (CV) for 30 min, washed with sterile water, and allowed to air dry for 10 min. Once dried, the CV was solubilized with absolute ethanol. The solution was transferred to a transparent 96-well plate, and the absorbance was quantified at 570 nm using a BioTek microplate reader. The experiments were performed in triplicate, and the data were represented as Mean ± SD for three experiments.

To observe the biofilm by SEM, biofilms of *A. baumannii* or *E. faecalis* were established and treated with CHU-1 as described above. Prior to CV staining, the biofilms in the wells were air-dried. Subsequently, the PVC wells were cut into small, smooth pieces, placed into petri dishes, and frozen at −80°C for 1 h. Following freezing, the samples underwent lyophilization for 5 h. The dried samples were then treated with metal spraying and observed using a Zeiss Gemini SEM 360 (Germany).

### 2.10 Antibacterial properties against persister cells

The antibacterial activity of CHU-1 against persister cells was carried out according to a previously established method with minor modifications (Mwangi et al., 2019). Ab YQ4 and Ef 10-17 were firstly cultured to the exponential phase and transferred to the PVC 96-well plate and incubated at 37°C for 24 h. To prepare persister cells, the planktonic bacteria were removed by washing three times with sterile 20 mM Tris–HCl (pH 7.4), followed by addition of 50× MIC of polymyxin B and linezolid for *A. baumannii* and *E. faecalis*, respectively. After incubation of 24 h, the planktonic bacteria were removed by triple washing with 20 mM Tris–HCl (pH 7.4). The attached persister cells were suspended in the same buffer. Then, 10 μM CHU-1 and ZnSO_4_ were added to the persister cell suspension and incubated at 37°C for 24 h, after which the number of viable bacteria was counted by gradient dilution and plating on the LB agar plate. No drug buffer was used as a negative control, and the experiments were conducted in triplicate. The data were represented as Mean ± SD for three experiments.

### 2.11 Determination of effect of temperature, pH, salt concentration and metal ions on the lytic activity of CHU-1

To evaluate the temperature stability of CHU-1, the enzyme was exposed to various temperatures (4°C–65°C) for 30 min. Subsequently, its lytic activity against *A. baumannii* was assessed in the reaction buffer at 37°C for 1 h, with the number of viable bacteria determined microbiologically. The reduced CFU at 37°C served as a reference, and the activity of CHU-1 at other temperatures was normalized to this reference activity.

To investigate the effects of pH on the activity of CHU-1, 10 μM of the enzyme was added to *A. baumannii* cells suspended in 20 mM Tris–HCl buffers with pH values ranging from 4.0 to 10.0. After incubation for 1 h, the number of viable bacteria was determined microbiologically. The reduced CFU at pH 7.4 was used as a reference, and the activity of CHU-1 at other pH levels was normalized to this reference activity.

To assess the impact of salt concentration on the activity of the engineering endolysin, 10 μM of the enzyme was added to *A. baumannii* cells suspended in 20 mM Tris–HCl buffer supplemented with 500 mM NaCl. ApoE23 was treated similarly. After incubation for 1 h, the number of viable bacteria was determined microbiologically. No salt buffer and 0.5 M NaCl were negative controls.

To assess the impact of different metal ions on the lytic activity of CHU-1, 10 μM of CHU-1 was added to *A. baumannii* and *E. faecalis* cells, respectively supplemented with 10 μM Zn^2+^, Ca^2+^ and Mg^2+^. No enzyme buffer was a negative control. CHU-1 without any metal ions was another control.

The experiments were conducted in triplicate. The data were represented as Mean ± SD for three experiments.

### 2.12 Synergy between endolysin and antibiotics

Antimicrobial interactions were assessed using the checkerboard assay (Odds, 2003). In a 96-well plate, CHU-1 solution was serially diluted along the abscissa, while antibiotics were diluted along the ordinate. Subsequently, the log-phase culture of *A. baumannii* or *E. faecalis* were added to the plate. Following incubation at 37°C for 24 h, OD_600_ was measured by a plate reader.

The fractional inhibitory concentration index (FICI) was then calculated for each combination using the formula (Odds, 2003): FICI = FIC(A) + FIC(B), where FIC(A) = MIC of drug A in combination/MIC of drug A alone, and FIC(B) = MIC of drug B in combination/MIC of drug B alone. Synergy was defined as a FICI ≤ 0.5, while a FICI between 0.5 and 1.0 indicated an additive effect. A FICI between 1.0 and 4.0 was considered indifferent, and a FICI > 4.0 suggested antagonism. Combinations demonstrating synergy were further confirmed in a 2 mL reaction system. The experiments were conducted in triplicate. The data were represented as Mean ± SD for three experiments.

### 2.13 Safety evaluation of CHU-1 *in vivo*

In this study, female SPF Kunming (KM) mice aged 8 weeks, weighing between 32 and 34 g, were obtained from the Animal Multiplying Farm of Qing Long Shan in Jiangning, Nanjing. Following a one-week acclimatization period with free access to food and water, the mice were housed under standard light/dark cycles (12 h of light and 12 h of darkness) at room temperature.

After the adaptation period, the mice were randomly divided into two groups, each comprising five mice. 200 μL of 0.5 mg/mL CHU-1 was administered to the KM mice via the intraperitoneal (IP) route. As a control, equal volume of 20 mM Tris–HCl buffer (pH 7.4) was used. Throughout the experiment, the mice’s clinical condition and body weights were monitored daily by two investigators for 2 weeks, starting from the time of injection. A scoring system adapted from the murine sepsis score criteria (MSS) was used (Shrum et al., 2014), with healthy mice assigned a score of 0 and deceased mice 5.

### 2.14 Bacteremia model of coinfection of *Acinetobacter baumannii* and *Enterococcus faecalis*

To establish a robust model for coinfection bacteremia, mice were intraperitoneally injected with various doses of log-phase cultures of Ab YQ4 and Ef 10-17. The high-dose group received a combination of 100 μL 1 × 10^9^ CFU/mL of Ab YQ4 and 100 μL 1 × 10^10^ CFU/mL of Ef 10-17 per mouse. Similarly, the middle-dose group was administered a combination of 100 μL 1 × 10^8^ CFU/mL of Ab YQ4 and 100 μL 1 × 10^9^ CFU/mL of Ef 10-17 per mouse, while the low-dose group received 100 μL 1 × 10^7^ CFU/mL of Ab YQ4 and 100 μL 1 × 10^8^ CFU/mL of Ef 10-17 per mouse. Each group comprised five mice. Over a 32-h period, two investigators meticulously monitored the clinical condition of the mice at various time points. The severity of sepsis in the mouse model was evaluated using a standardized scoring system previously described (Shrum et al., 2014), which assessed appearance, level of consciousness, activity, and response to stimuli, eye and respiration rate. Healthy mice were assigned a score of 0, while deceased mice were assigned a score of 5.

### 2.15 Treatment of coinfection bacteremia by CHU-1

For treatment, mice were randomly divided into three groups, with 8 mice each group. To assess the protective efficacy of CHU-1, a high dose of the bacterial mixture was prepared as described earlier and administered to the mice via intraperitoneal injection. At 0.5 h post-infection (POI), intraperitoneal injections of 200 μL of 0.5 mg/mL CHU-1 were administered. Prior to use, equal molar concentration of Zn^2+^ was premixed with CHU-1. Mice without infection served as the negative control group. As a non-treatment control, sterile 20 mM Tris–HCl buffer (pH 7.4) was used to treat infected mice. Subsequently, the survival rates were monitored at various time points, and the survival curve was analyzed using the Kaplan–Meier method with GraphPad Prism 9.3.0.

To further investigate the bactericidal efficacy of CHU-1 *in vivo*, mice were randomly assigned to three groups, with 5 mice per group. The middle dose of the bacterial mixture was prepared as described earlier and administered to the mice via intraperitoneal injection. At 0.5 h POI, the same treatment regimen was administered intraperitoneally. After 6 h, the mice were euthanized, and retro-orbital bleeding was performed to collect blood for bacterial counting. Additionally, the livers and spleens of the euthanized mice were harvested for bacterial counting.

### 2.16 Statistical analysis

Statistical analysis was performed using GraphPad Prism software 9.3.0. The statistical differences among different groups were assessed by One-way ANOVA (nonparametric or mix) with multiple comparison.

## 3 Results

### 3.1 Phage Kp84B lysed the pandrug-resistant *Klebsiella pneumoniae* Kp84

**Figure 1:**
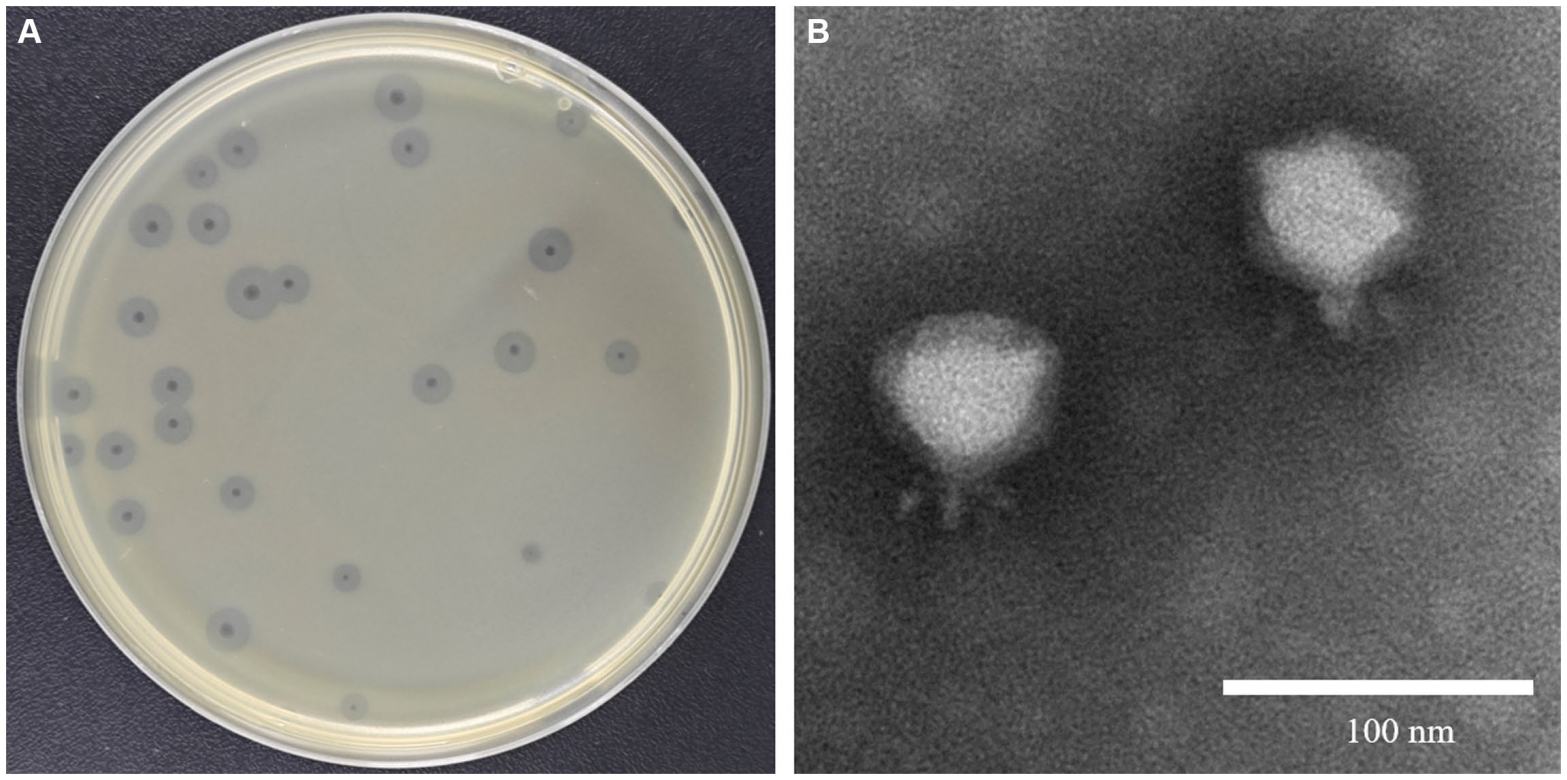
Isolation and characterization of ФKp84B **(A)** ФKp84B formed big and clear plaques with halo rings against the pandrug-resistant strain Kp 84. **(B)** TEM observation showed that ФKp84B possessed a short tail, belonging to the Podoviridae family.

**Figure 2:**
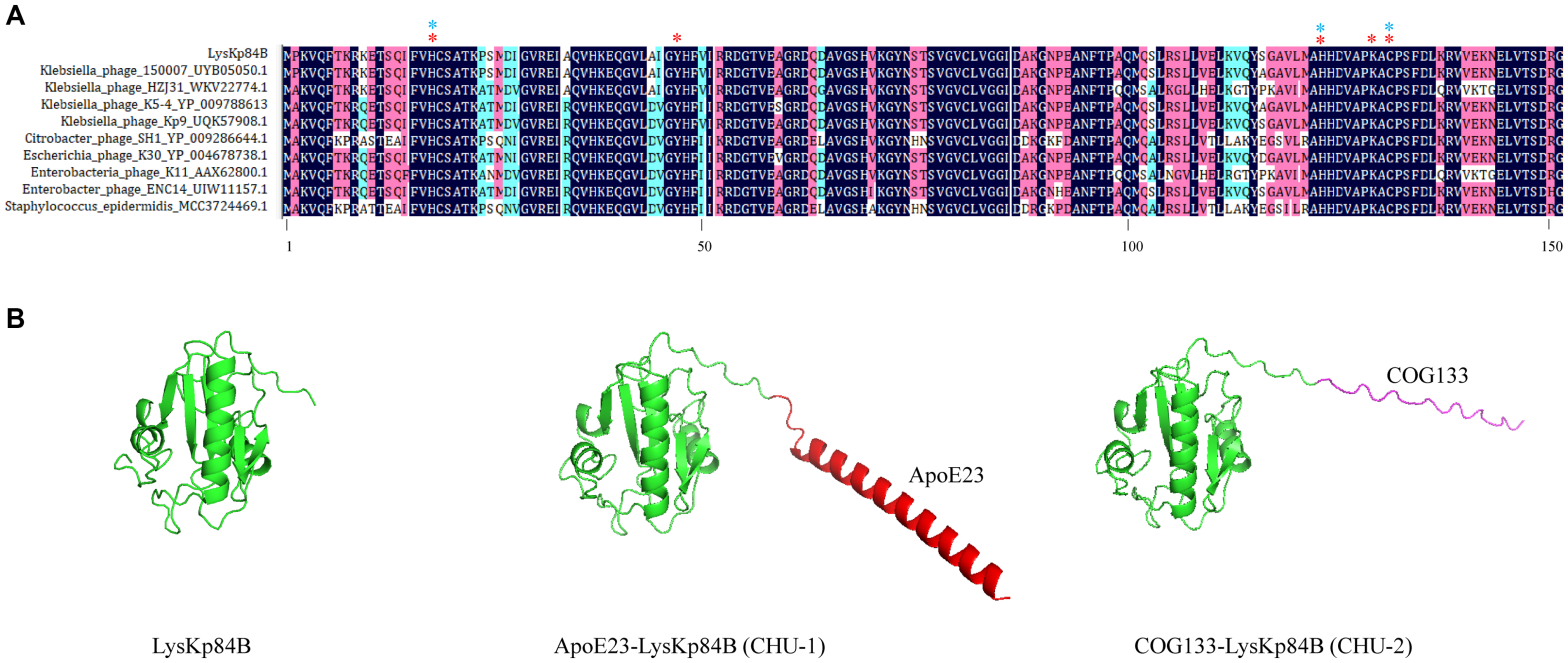
Multiple sequence alignment of LysKp84B and 3D structures of its derivatives. **(A)** Multiple sequence alignment of LysKp84B by DNAman software. The protein sequences were from BLASTp analysis using LysKp84B as query. Red stars indicate active sites. Blue stars indicate residues involved in Zinc ion binding. **(B)** Protein structures of LysKp84B, CHU-1 and CHU-2, predicted by Alphafold v2.3.2. All the parameters were default.

*Klebsiella pneumoniae* 84 (Kp 84) was a clinically isolated pandrug-resistant strain in our hospital, which contained a circular chromosome of 5,412,390 bp with GC content 50.1% and 4 circular plasmids. MLST analysis showed that KP84 belonged to ST11, which was among the most important clinical pathogens in China (Zhao et al., 2020). From the untreated hospital waste water, we isolated a lytic phage against Kp84, which formed big and clear plaques, and therefore were named as ФKp84B (Figure 1A). TEM observation revealed an icosahedron head and a short tail (Figure 1B), indicating that ФKp84B belonged to Caudoviricetes. This phage possessed a circular dsDNA genome of 40,574 bp, with GC content 52.38%. Blast analysis with the whole genome sequence as query showed that the most similar phage was *Klebsiella phage* SH-Kp 152,410 (NC_047908.1) isolated in Shanghai, with 97% coverage and 97.51% identity. ФKp84B was composed of 50 genes, including 19 genes involved in packaging, replication and assembly, 9 genes involved in lysis and infection, 2 genes involved in regulation and 20 genes with unknown function. ФKp84B had an endolysin of 151 amino acids, 16.8 kDa, and named as LysKp84B. Blast analysis revealed that LysKp84B is an amidase. Interestingly, multiple sequence alignment analysis demonstrated high similarity between the protein sequence of LysKp84B and endolysins originating from various bacterial phages, such as *Klebsiella*, *Escherichia*, *Citrobacter*, *Enterobacter*, and *Staphylococcus* (Figure 2A). The conservation of this sequence suggests that LysKp84B may possess broad lytic activity against multiple bacterial species.

### 3.2 LysKp84B itself possesses weak bactericidal activity

**Figure 3:**
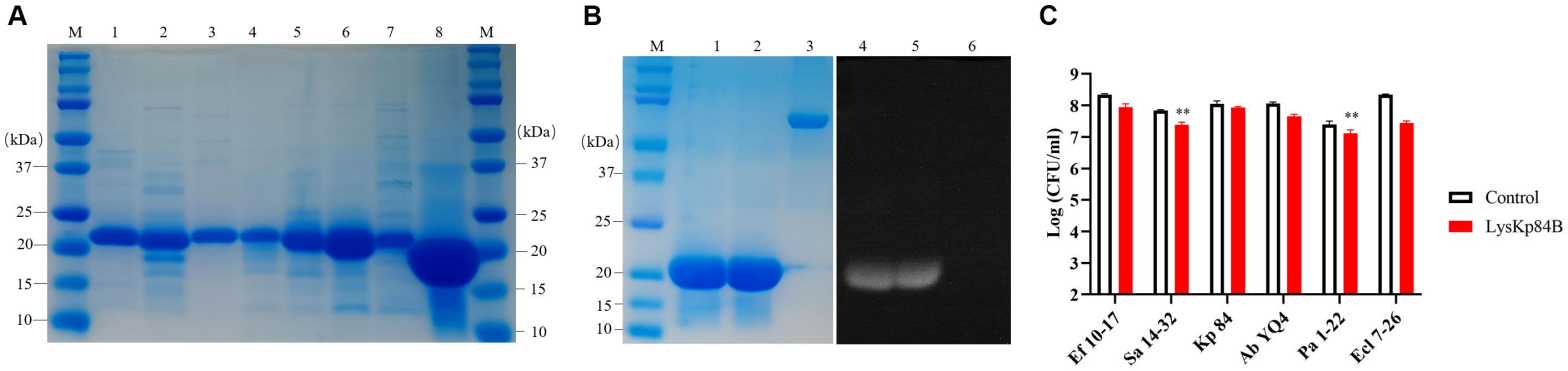
Purification of recombinant proteins and determination of bactericidal activity of LysKp84B. **(A)** SDS-PAGE of recombinant proteins. Lane 1, CHU-1; lane 2, CHU-2; lane 3, COG133-L4-Kp84B; lane 4, COG133-L3-Kp84B; lane 5, COG133-L2-Kp84B; lane 6, COG133-L1-Kp84B; lane 7, COG133-Kp84B (Y47A); Lane 8, LysKp84B. **(B)** Zymography analysis of LysKp84B. Lane 1 and lane 4, LysKp84B; lane 2 and lane 5, LysKp84B with Zn^2+^; lane 3 and lane 6, BSA. **(C)** Dynamic killing of LysKp84B against “ESKAPE” strains *in vitro*. No endolysin addition served as negative control.

His-tagged LysKp84B was purified through standard immobilized metal affinity chromatography (IMAC) method (Figure 3A). In the zymographic assay, LysKp84B exhibited peptidoglycan hydrolytic activity, independent of zinc ions (Figure 3B). However, in terms of bactericidal capability, LysKp84B only demonstrated weak activity against carbapenem-resistant *E. cloacae* strain 7-26 (Ecl 7-26) and methicillin-resistant *Staphylococcus aureus* strain 14–32 (Sa 14-32), showing no significant effect on strain Kp 84, pandrug-resistant *Acinetobacter baumannii* strain YQ4 (Ab YQ4), carbapenem-resistant *Pseudomonas aeruginosa* strain 1-22 (Pa 1-22), or vancomycin-resistant *Enterococcus faecium E. faecalis* (Ef 10-17; Figure 3C). These results indicate that LysKp84B has very limited bactericidal activity *in vitro*.

### 3.3 Engineering LysKp84B via fusing membrane-penetrating peptides

To enhance the potency of LysKp84B, we opted to fuse it with membrane-penetrating peptides, ApoE23 or COG133. Both peptides are short cationic sequences derived from ApoE, known for their robust antimicrobial activity through the disruption of cell membrane integrity (Wang et al., 2013; Pane et al., 2016). The predicted structures of the fusion proteins, as determined by AlphaFold2, are illustrated in Figure 2B. LysKp84B is a compact single-domain globular protein with a molecular weight of 16.8 kD, which displayed distinct structural alterations when fused with ApoE23 or COG133. Fusion with ApoE23 resulted in an alpha helix at the N-terminus of LysKp84B, while fusion with COG133 led to an extended coil at the N-terminus. These engineered proteins were designated as CHU-1 and CHU-2, respectively.

### 3.4 Engineered endolysins exhibits enhanced bactericidal activity and extended antimicrobial spectrum

**Figure 4:**
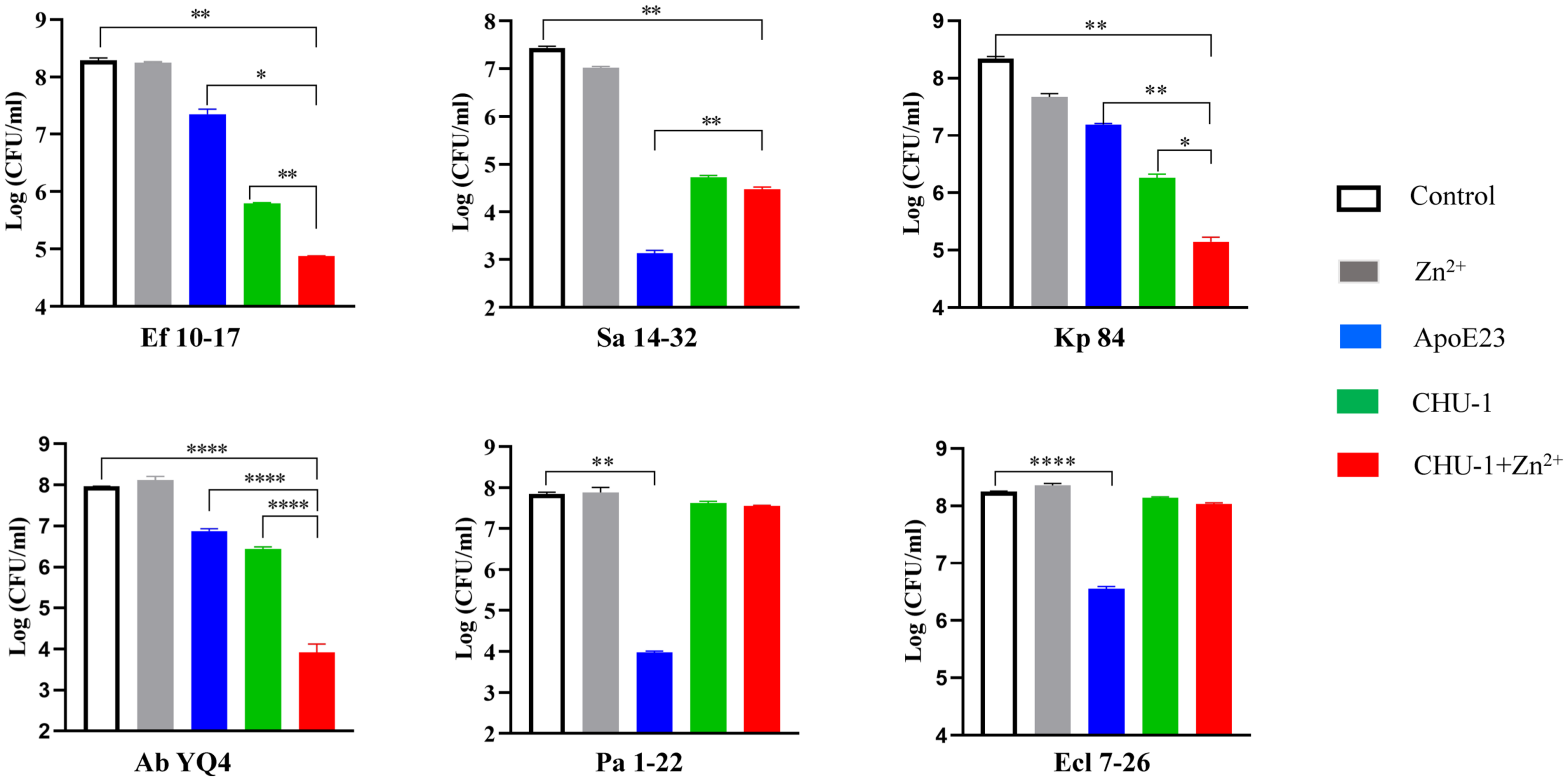
Dynamic killing of CHU-1 against “ESKAPE” strains. CHU-1 was incubated with “ESKAPE” strains and the CFU was counted through standard dilution and plating. One-way ANOVA with multiple comparisons was used to analyze the differences among different groups. * indicates *p* < 0.05; ** indicates *p* < 0.01; *** indicates *p* < 0.001; **** indicates *p* < 0.0001.

**Figure 5:**
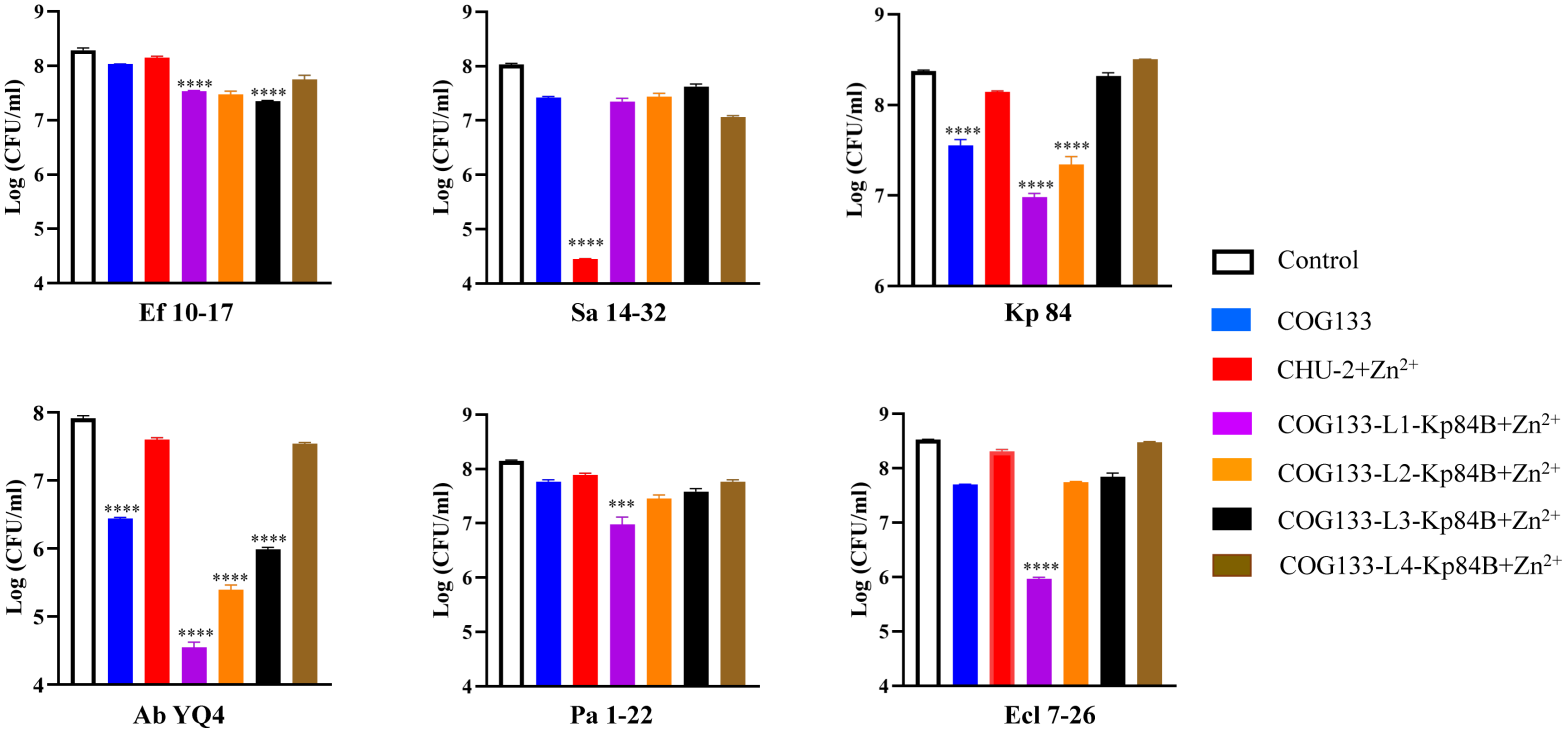
Dynamic killing of CHU-2 and its derivatives against “ESKAPE” strains. CHU-2 or its derivatives were, respectively, incubated with “ESKAPE” strains and the CFU was counted through standard dilution and plating. One-way ANOVA with multiple comparisons was used to analyze the differences among different groups. * indicates *p* < 0.05; ** indicates *p* < 0.01; *** indicates *p* < 0.001; **** indicates *p* < 0.0001.

To evaluate the bactericidal effectiveness of ApoE23-Kp84B (CHU-1), we quantified the reduction in colony-forming units (CFU) for the six bacterial strains following exposure to CHU-1 (Figure 4). Initially, to eliminate the influence of zinc ions on bactericidal activity, we determined the minimum inhibitory concentration (MIC) of zinc ions alone for these six strains, finding MICs above 1.5 mM (Supplementary Table S3). The addition of 10 μM zinc ions had minimal impact on CFU reduction. As expected, ApoE23 alone demonstrated a broad bactericidal spectrum, particularly against *S. aureus* and *P. aeruginosa*. Notably, CHU-1 surpassed ApoE23 in killing efficacy against *E. faecalis*, *K. pneumoniae*, and *A. baumannii*. This advantage was further accentuated with the inclusion of zinc ions. Within 1 h, 10 μM CHU-1 reduced CFU by over 3 log units for these three strains. Conversely, the fusion of ApoE23 and Kp84B compromised the bactericidal capacity against *S. aureus*, *P. aeruginosa*, and *E. cloacae*, compared with ApoE23 alone. On the other hand, COG133 alone demonstrated significant bactericidal activity exclusively against *K. pneumoniae* and *A. baumannii*. However, when fused with LysKp84B, CHU-2 exhibited remarkable killing efficiency against *S. aureus*, resulting in a reduction of over 3 log units of CFU within 1 h in the presence of zinc ions (Figure 5). Considering the key role of zinc ions in the bactericidal activity of endolysin, zinc ions were added in the following experiments involved in endolysin. In summary, our engineering approach successfully enhanced the antimicrobial activity of LysKp84B and broadened its antimicrobial spectrum.

### 3.5 Insertion of one GS linker enhances bactericidal activity

To investigate the impact of linkers between COG133 and LysKp84B, we empirically selected the widely used flexible “GS linker,” composed of (Gly-Gly-Gly-Gly-Ser)_n_ (Chen et al., 2013). Examining the effect of different linker lengths on the antimicrobial activity of the engineered protein, we, respectively, inserted 1, 2, 3, and 4 GS linkers into CHU-2. Notably, the insertion of one linker in COG133-Kp84B (COG133-L1-Kp84B) showed the most significant enhancement in bactericidal capacity, particularly against *A. baumannii* and *E. cloacae*, resulting in 3 and 2 log unit reductions of CFU, respectively. Meanwhile, CHU-3 also exhibited outstanding bactericidal activity against *P. aeruginosa* and *K. pneumoniae*, causing over 90% reduction within 1 h in the presence of zinc ions (Figure 5). The insertion of 4 GS linkers did not enhance the efficacy of CHU2, implying that proximity between the membrane-penetrating peptide and endolysin is crucial for synergistic action.

### 3.6 Treatment of engineered endolysins changes bacterial morphologies

**Figure 6:**
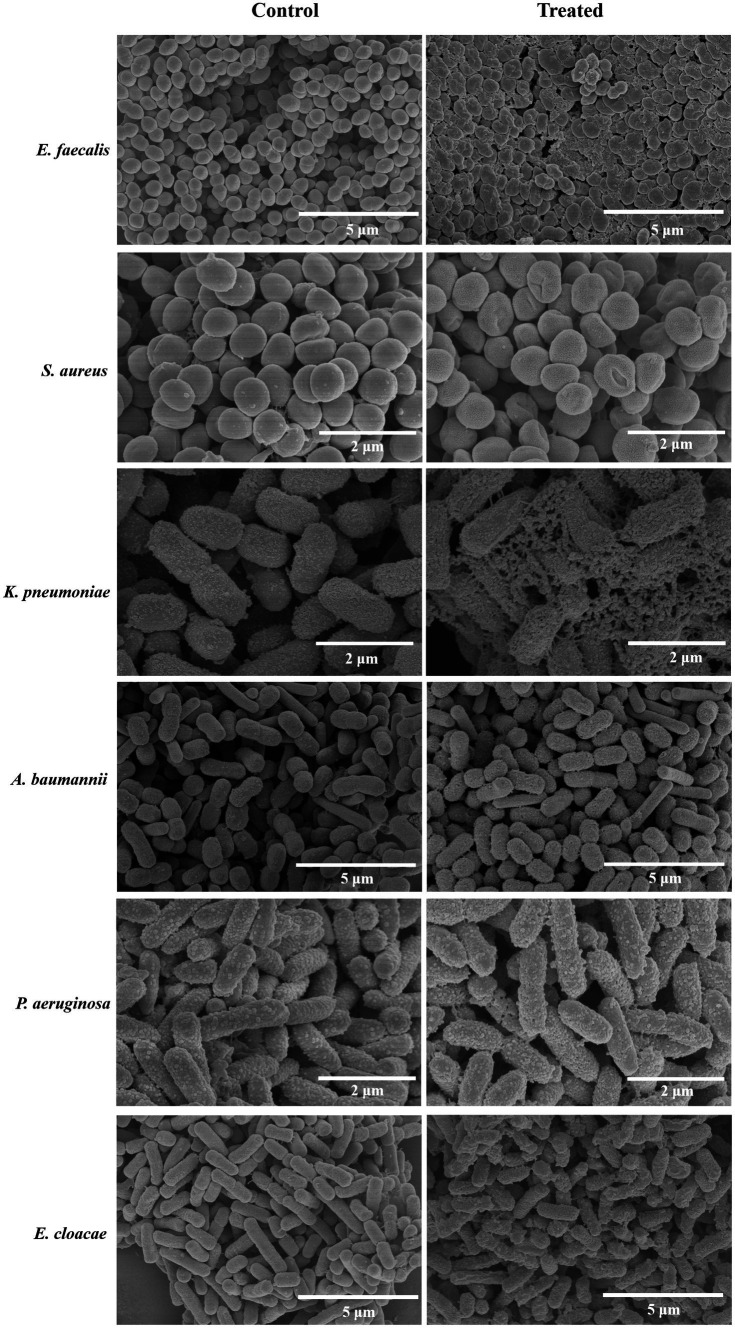
SEM images of ESKAPE bacteria treated by engineered endolysins. Scanning electron microscopy was utilized to observe the morphological changes in ESKAPE bacteria following treatment with engineered endolysins. *Acinetobacter baumannii*, *Enterococcus faecalis*, and *Klebsiella pneumoniae* were treated with CHU-1, while *Staphylococcus aureus* was treated with CHU-2. Additionally, *Enterobacter cloacae* and *Pseudomonas aeruginosa* were treated with COG133-L1-Kp84B. The untreated bacteria served as the negative control.

To elucidate the mechanism of action of the engineered endolysins, we examined the surface morphologies of treated bacteria using scanning electron microscopy (SEM; Figure 6). Following exposure to CHU-1, the surface of *A. baumannii* became noticeably rough, with the emergence of numerous tiny holes. *Enterococcus faecalis* exhibited distorted shapes, with many cells visibly lysed, while *K. pneumoniae* appeared fragmented into pieces. Upon treatment with CHU-2, *S. aureus* underwent a transformation from a “plump and slippery grape” appearance to a “rough and deflated waxberry” morphology. Notably, COG133-L1-Kp84B treatment induced a crispy and distorted appearance in *P. aeruginosa* and *E. cloacae*, accompanied by the presence of tiny holes on their surfaces. In summary, the engineered endolysins induced alterations in bacterial morphologies, aligning with their known targets on the cell membrane and peptidoglycan in the cell wall.

### 3.7 CHU-1 treatment increases permeabilization of cell membranes

**Figure 7:**
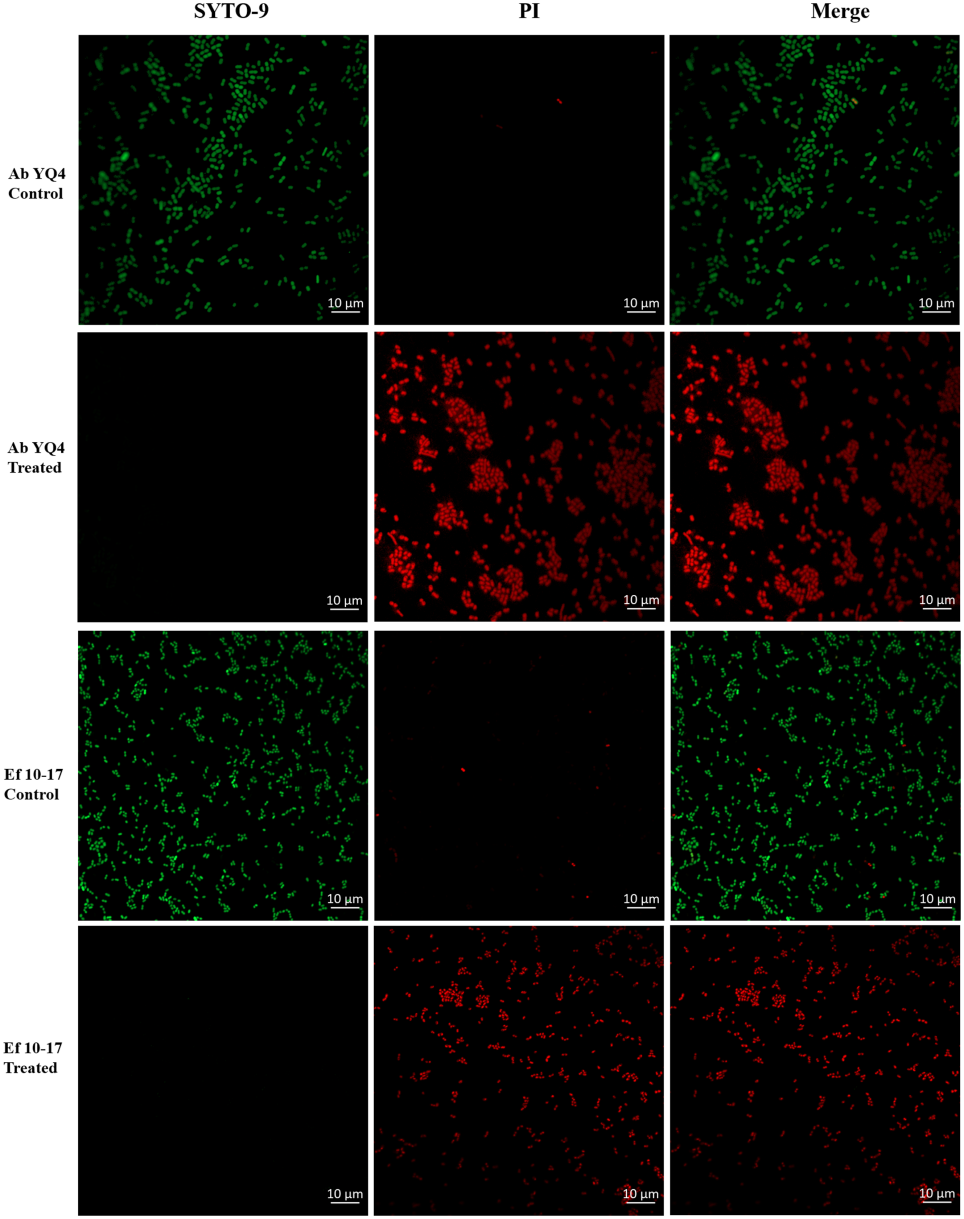
Treatment of CHU-1 impairs bacterial membrane integrity. Ab YQ4 and Ef 10-17 were treated by CHU-1, stained by SYTO-9 and PI, and observed by confocal fluorescence microscopy. Bacteria without treatment served as controls. Scale bar: 10 μm.

Given the propensity of *E. faecalis* to contribute to polymicrobial infections (Xu et al., 2024), our focus shifted to evaluating CHU-1’s efficacy against both *A. baumannii* and *E. faecalis*, as well as their potential coinfection. Initially, we investigated whether CHU-1 treatment resulted in increased permeabilization of the plasma membrane. To accomplish this, CHU-1-treated cells were subjected to staining with two fluorescent nucleic acid dyes, SYTO-9 and propidium iodide (PI). As anticipated, untreated controls exhibited viable cells, as evidenced by green fluorescence upon SYTO-9 staining. In contrast, CHU-1-treated cells exhibited red fluorescence upon PI staining (Figure 7), indicating bacterial cell death and heightened membrane permeabilization following CHU-1 treatment.

### 3.8 CHU-1 treatment efficiently eradicates mature biofilms and persister cells

**Figure 8:**
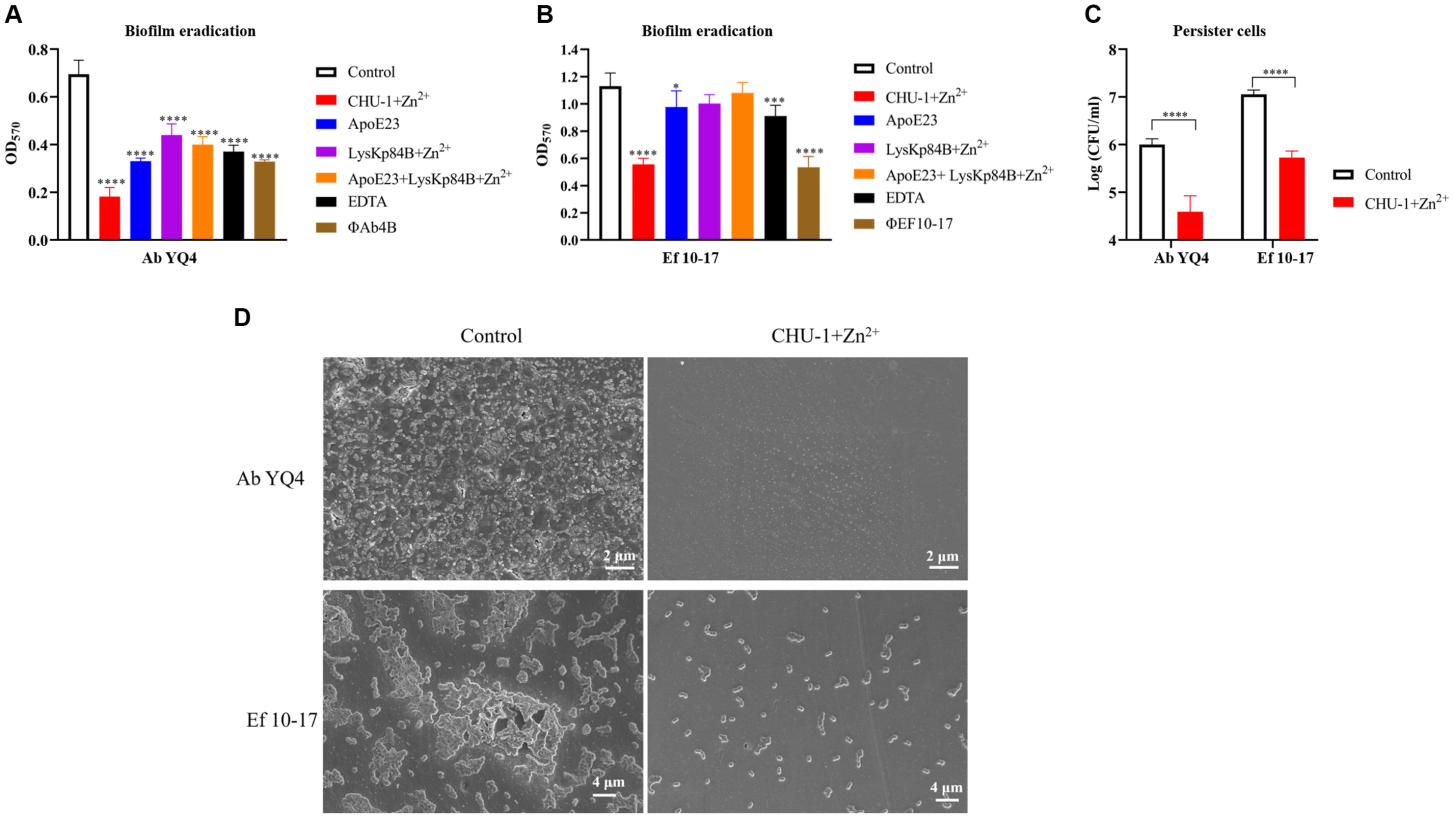
CHU-1 displays potent activity of anti-biofilm and anti-persister cell. CHU-1 was efficiently able to eradicate mature biofilms of *Acinetobacter baumannii* **(A)** and *Enterococcus faecalis* **(B)** in the presence of Zn^2+^. Antimicrobial peptides, EDTA and phages were used positive controls. No drug treatment served as negative control. The combination use of antimicrobial peptide and LysKp84B was also analyzed. **(C)** CHU-1 displays potent activity against the persister cells of *A. baumannii* and *E. faecalis*. **(D)** SEM images of biofilms of *A. baumannii* and *E. faecalis* treated or untreated by CHU-1, with equal molar Zn^2+^ supplemented.

Biofilm formation represents a critical virulence factor for bacterial pathogens and often poses a challenge in clinical settings due to its resilience. To assess the biofilm eradication potential of CHU-1, we initiated by cultivating mature biofilms for 24 h, followed by treatment with CHU-1. In the case of *A. baumannii*, partial removal of the biofilm was achieved using the antimicrobial peptide ApoE23, EDTA, as well as phage ФAb4B, which is a lytic phage against Ab YQ4 and exhibits favorable anti-biofilm activity. Intriguingly, CHU-1 demonstrated superior efficacy compared to all these agents. Notably, CHU-1 exhibited even greater potency than the combination of ApoE23 and LysKp84B (Figure 8A). Similar trends were observed for *E. faecalis*, where CHU-1 matched the effectiveness of the anti-*E. faecalis* phage ФEF10-17 (Figure 8B). Remarkably, CHU-1 was capable of eliminating one log unit of persister cells of *A. baumannii* or *E. faecalis* within 24 h (Figure 8C).

To further evaluate the effect of CHU-1 on clearing biofilm, we observed the biofilms of *A. baumannii* and *E. faecalis* by scanning electron microscopy (SEM). Consistently, under CHU-1 treatment, their mature biofilms were almost eradicated (Figure 8D). Collectively, these findings underscore CHU-1 as a promising agent for combating biofilms and persister cells.

### 3.9 CHU-1 exhibits synergy with polymyxin B against *Acinetobacter baumannii*

In LB broth, CHU-1 exhibited a minimal inhibitory concentration (MIC) of 12 μM for *A. baumannii* YQ4 and 24 μM for *E. faecalis* 10–17. When combined with polymyxin B, CHU-1 showed significant synergy, reflected by a fractional inhibitory concentration (FIC) index of 0.15. Notably, the combination of 1/32 MIC CHU-1 and 1/8 MIC polymyxin B completely inhibited the growth of *A. baumannii* YQ4 in LB broth (Supplementary Figure S1). Furthermore, CHU-1 displayed an additive effect when combined with Ciprofloxacin and Meropenem, with FIC values of 0.5 and 1.0, respectively. However, no additive or synergistic effect of CHU-1 with conventional antibiotics against *E. faecalis* was observed.

### 3.10 CHU-1 exhibits high resistance against high salt

Considering the pivotal role of the membrane-penetrating peptide in the bactericidal activity of the engineered endolysin, and recognizing that high salt levels can interfere with the function of cationic peptides (Kandasamy and Larson, 2006), we conducted experiments to determine whether CHU-1 functions normally in a high-salt buffer. Interestingly, we found that CHU-1 retained its ability to kill *A. baumannii* and *E. faecalis* even in the presence of 0.5 M NaCl, exhibiting potentially increased potency compared to a normal buffer (Supplementary Figures S2A,B).

Furthermore, we characterized the heat and pH tolerance of CHU-1. Our findings revealed that CHU-1 behaved consistently across a range of temperatures from 4°C to 37°C and pH values from 4.0 to 9.0. However, CHU-1’s efficacy diminished rapidly at temperatures exceeding 45°C and pH levels exceeding 10 (Supplementary Figures S2C,D). We also determined the effects of divalent metal ions on CHU-1’s enzymatic activity and found that only Zn^2+^ could enhance CHU-1’s bacterial activity, but not Ca^2+^ or Mg^2+^ (Supplementary Figure S2E).

### 3.11 CHU-1 demonstrates safety *in vivo*, but falls short in mouse bacteremia model

**Figure 9:**
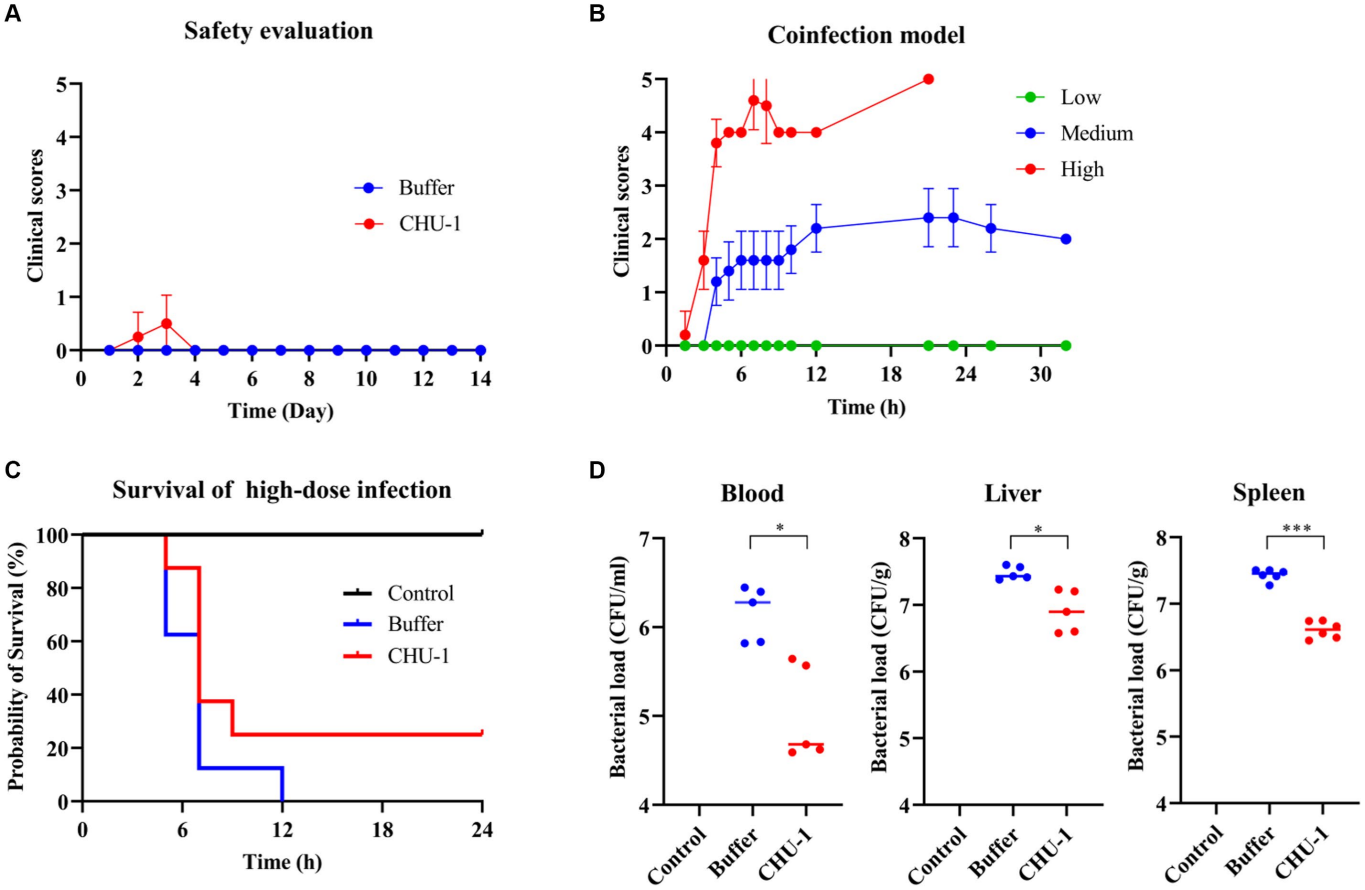
CHU-1 is safe *in vivo* but falls short in the protective efficacy for bacteremia mouse model. **(A)** Clinical scores of mice administrated with CHU-1. Tris–HCl buffer was used as a control. **(B)** Bacteremia mouse model of coinfection of *Acinetobacter baumannii* and *Enterococcus faecalis* with low, middle and high dose. **(C)** CHU-1 treatment saved 25% infected mice in the high-dose coinfection model. **(D)** CHU-1 treatment reduced bacterial loads in bloodstream and organs in the middle-dose coinfection model. One-way ANOVA with multiple comparisons was used to analyze the differences among different groups. * indicates *p* < 0.05; *** indicates *p* < 0.001.

To evaluate the safety of CHU-1, 200 μL of 0.5 mg/mL CHU-1 was administered to the KM mice via the intraperitoneal (IP) route. Clinical scores were monitored at various time intervals over a period of 14 days. The administration of CHU-1 did not result in any significant clinical effects (Figure 9A). There was no significant difference with respect to the body weights (data not shown). Subsequently, to assess the antimicrobial efficacy of CHU-1 *in vivo*, we established a bacteremia mouse model induced by coinfection of *A. baumannii* and *E. faecalis*. Mice were inoculated with different doses of the bacterial mixture via the IP route. High bacterial doses led to mortality within 24 h, while medium doses caused mild symptoms (Figure 9B). Initially, we evaluated the efficacy of CHU-1 in mice infected with a high dose of the bacterial mixture. Unfortunately, only 25% (2/8) of the mice survived at 24 h post-infection (POI; Figure 9C). Subsequently, we infected mice with a mild dose of the bacterial mixture and euthanized them after 6 h POI to determine bacterial loads in blood and organs. We observed that CHU-1 significantly reduced bacterial loads, albeit by less than one log unit (Figure 9D). At 24 h POI, approximately 10^5^ CFU/mL of bacteria remained in the blood of mice treated with CHU-1. In summary, CHU-1 demonstrated safety at this dosage, but its bactericidal efficacy was severely compromised *in vivo*.

## 4 Discussion

Phage-originated lytic enzymes, including endolysins and capsular polysaccharide depolymerases, are significant reservoirs for antimicrobial agents (Knecht et al., 2020; Rahman et al., 2021). While phages typically demonstrate highly specific host ranges, endolysins exhibit a broader spectrum compared to their phage counterparts. They not only target a greater variety of strains within a single species but also demonstrate activity across different genera. For example, LysPBC2, a natural endolysin derived from a *Bacillus cereus* phage, exhibited lytic activity against *Bacillus*, *Listeria*, and *Clostridium* species (Yoong et al., 2004). It’s noteworthy that endolysin applications primarily focus on Gram-positive bacteria due to the peptidoglycan layer’s external position in their cell walls (Murray et al., 2021). This external positioning facilitates easier interaction between the endolysin and its substrate within these bacteria. In contrast, Gram-negative bacteria have a more intricate cell wall structure comprising an outer membrane, peptidoglycan layer, and inner membrane. The outer membrane poses a significant barrier to endolysin access to the peptidoglycan layer. Consequently, most reported Gram-negative endolysins exhibit weak antimicrobial activity in the absence of membrane permeabilizers (Rahman et al., 2021). Consistently, our study found that LysKp84B displayed weak bactericidal activity against *K. pneumoniae*. To our surprise, it also showed limited efficiency in killing Gram positive bacteria like *E. faecalis* and *S. aureus*, even in the presence of Zn ions. This observation is intriguing because Gram-positive bacteria naturally expose their peptidoglycan layer, which endolysins should be able to access freely. The low killing efficacy might be due to the cover of capsular polysaccharide (Hancock and Gilmore, 2002; Chan et al., 2014). Moreover, when fused with membrane-penetrating peptides, CHU-1 and CHU-2 exhibited activity against both Gram-negative and Gram-positive bacteria. How does the peptide sequence help endolysin? A plausible explanation is that the fused antimicrobial peptide facilitates the disruption of the inner membrane. The underlying mechanism warrants further investigation.

The efficacy of endolysins is subject to various influencing factors, encompassing amino acid composition, polarity, charge distribution, and overall structural configuration (Plotka et al., 2019; Gallego-Páramo et al., 2021; Liu et al., 2023). Modifying endolysins entails manipulating these factors to optimize their activity against bacteria. One particularly intriguing strategy involves fusing endolysins with antimicrobial peptides to augment their capacity to penetrate the cell walls of Gram-negative bacteria. For instance, Islam et al. (2022) demonstrated significant enhancement in the bactericidal activity of the chimeric endolysin against multidrug-resistant *A. baumannii* isolates by fusing cecropin A, a commonly used antimicrobial peptide, with the N-terminus of Ab endolysin. Similarly, PaAmi1, an amidase from *Propionibacterium* bacteriophage PAC1, exhibited an expanded lytic spectrum when fused with two antimicrobial peptides (Varotsou et al., 2023). In our study, we opted to fuse endolysins with two antimicrobial peptides derived from ApoE, COG133, and ApoE23. Even though they have different lengths and amino acid compositions, both peptides share a common feature, several positively charged amino acids, such as lysine (K) or arginine (R). Notably, previous research indicates that increasing the net charge of endolysins, such as through the incorporation of positively charged amino acids, can enhance their bactericidal effectiveness. This has been exemplified by the development of artilysins containing positively charged amino acids at one end (Carratalá et al., 2023). Additionally, Sui et al. (2023) found that randomly fusing short peptides with endolysin resulted in chimeric endolysins with extracellular activities, characterized by a peptide sequence featuring a positive charge and an α-helical structure. However, the precise differences between ApoE23 and COG133 that account for the divergent activity and lytic spectrum observed between CHU-1 and CHU-2 remain unclear. Further investigation is warranted to elucidate the key determinants of the lytic activity of engineered endolysins and to unravel the nuances underlying their interactions with antimicrobial peptides.

The GS linker, composed of glycine and serine residues arranged in a repeated pattern, is widely recognized for its flexibility and ability to maintain the functional integrity of fused protein domains (Ceballos-Alcantarilla and Merkx, 2021). The decision to empirically test different linker lengths was driven by the need to optimize the spatial arrangement between the COG133 and LysKp84B domains within the engineered protein. We hypothesized that varying the number of GS linkers would influence the flexibility and orientation of the fused domains, thereby impacting their antimicrobial activity. Interestingly, our results revealed that the insertion of a single GS linker in COG133-Kp84B (COG133-L1-Kp84B) led to the most significant enhancement in bactericidal capacity, particularly against *A. baumannii* and *E. cloacae*. We attribute this enhancement to several factors. Firstly, the insertion of a single GS linker may provide sufficient flexibility to optimize the spatial arrangement between the COG133 and LysKp84B domains, allowing for improved interaction with target bacterial cells. Additionally, the presence of a single linker may minimize steric hindrance and maintain the structural integrity of the engineered protein, enabling efficient membrane penetration and subsequent bactericidal activity. In contrast, the insertion of additional GS linkers did not further enhance the efficacy of the engineered protein, suggesting that an optimal spatial arrangement between the membrane-penetrating peptide and endolysin domain is crucial for synergistic action. Excessive flexibility conferred by multiple linkers may disrupt the proper orientation of the fused domains, leading to diminished antimicrobial activity. Overall, our findings underscore the importance of linker design in optimizing the function of engineered proteins and highlight the intricate balance between flexibility and structural integrity in achieving enhanced antimicrobial activity.

Endolysins exhibit significant diversity in functions (Oechslin et al., 2022). LysKp84B, annotated as an amidase, functions by cleaving the amide bond between the lactyl group of muramic acid and the amino group of L-alanine to release a peptide moiety (Lupoli et al., 2009). Intriguingly, LysKp84B shares homologous proteins conserved among various bacterial phages, including *Escherichia*, *Citrobacter*, *Enterobacter*, and *Staphylococcus*. This observation suggests a potential common ancestry for these endolysins. Moreover, this shared similarity may elucidate why LysKp84B demonstrates the ability to degrade the peptidoglycan layer of these bacteria, particularly with the assistance of antimicrobial peptides. Interestingly, we exclusively observed a synergistic interaction between CHU-1 and polymyxin B, but not with other antibiotics, despite their distinct mechanisms of action from endolysins. Notably, polymyxins primarily target bacterial membranes, disrupting their integrity and inducing cell death, rendering them as last-resort antibiotics for Gram-negative bacteria (Briers et al., 2014). This finding aligns with the study by Sitthisak et al. (2023), which reported a synergistic interaction between an engineered endolysin from phage vB_AbaM_PhT2 and colistin against *A. baumannii*, with a FICI of 0.25. Similarly, the engineered EC340, derived from phage PBEC131 infecting *Escherichia coli*, demonstrated a synergistic effect exclusively with colistin and an additive effect with meropenem, tigecycline, chloramphenicol, azithromycin, and ciprofloxacin (Hong et al., 2022). On the other hand, in our study, the observed synergistic effect between CHU-1 and polymyxin B was specific to *A. baumannii*, with no such effect observed for the Gram-positive bacterium *E. faecalis*. This observation suggests that the key determinant of synergy lies in the disruption of the outer membrane by polymyxin B, thereby facilitating the entry of endolysins. Other antibiotics with membrane-disrupting properties could be explored in the future, to provide a comprehensive understanding of their effects and potential synergies with engineered endolysins.

In the context of endolysins, there is interaction between metal ions and enzymatic activity. For example, Psa, a zinc-dependent amidase endolysin, demonstrated lytic activity against *C. perfringens* under physiological conditions. Interestingly, the addition of excess zinc ions resulted in a concentration-dependent decrease in Psa’s lytic activity (Sekiya et al., 2021). Another example is LysB4, an endolysin from the *Bacillus cereus*-infecting bacteriophage B4 (Son et al., 2012). Its enzymatic activity depends on divalent metal ions. Removal of metal ions using EDTA significantly decreased LysB4’s lytic activity, indicating the requirement of metal ions for full enzymatic function. Addition of Zn^2+^ or Mn^2+^ restored LysB4’s activity, highlighting the importance of these specific divalent metal ions. In contrast, other divalent metal ions such as Ca^2+^ and Mg^2+^ could restore enzymatic activity only at higher concentrations. In our study, we also found that the same concentration of Ca^2+^ and Mg^2+^ was not as potent as Zn^2+^ to enhance CHU-1’s activity. Overall, our findings underscore the intricate relationship between zinc ions and endolysin activity, with implications for their therapeutic potential. Further research is warranted to elucidate the precise mechanisms underlying these interactions and optimize endolysin formulations for therapeutic applications.

The study’s limitation lies in the modest protective efficacy of CHU-1 against bacteremia in mice, reaching only 25%. There are two primary contributing factors to this outcome. Firstly, our coinfection model employed a high-dose mixture of *A. baumannii* and *E. faecalis*, resulting in excessively severe symptoms and rapid mortality. The administration of CHU-1 at 0.5 h post onset of infection may have been too delayed to effectively rescue the infected mice. Secondly, since CHU-1 is expressed in *E. coli*, the achievable final concentration is constrained, remaining below 1 mg/mL. Consequently, the production yield falls short, further exacerbated by the loss of recombinant proteins during endotoxin removal. As a result, the treatment site may not have received an adequate dosage of endolysins. Exploring the expression of endolysins in *Bacillus subtilis*, devoid of endotoxins, warrants consideration for future investigations. Moreover, systemic administration of endolysins encounters a notable hurdle, proteases present in the bloodstream. Previous reports indicate that eAbEndolysin achieved a 40% survival rate in mice with systemic *A. baumannii* infection (Islam et al., 2022), while PlyGRCS rescued 30% of mice from *S. aureus* bacteremia-induced mortality (Linden et al., 2015). Notably, our study represents the first instance of employing an engineered endolysin to address a coinfection sepsis model. Our subsequent plan involves investigating the topical application of our engineered endolysins for the for treatment polymicrobial infection of ESKAPEE, aiming to address these challenges.

In a conclusion, we successfully engineered a natural endolysin derived from an isolated *K. pneumoniae* phage by fusing two ApoE mimetic peptides, ApoE23 and COG133, at the N-terminus of the endolysin. ApoE23-Kp84B (CHU-1) reduced over 3 log units of CFU for *A. baumannii*, *E. faecalis*, *K. pneumoniae* within 1 h, while COG133-Kp84B (CHU-2) showed significant efficacy against *S. aureus*. COG133-L1-Kp84B, with a GS linker insertion in CHU-2, exhibited outstanding bactericidal activity against *E. cloacae* and *P. aeruginosa*. This innovative approach significantly enhanced the bactericidal activity against planktonic cells, biofilm and persister cells. Moreover, the engineered endolysins exhibited a broader antimicrobial spectrum, demonstrating activity against all tested “ESKAPEE” strains. Despite these advancements, challenges persist, including optimizing delivery modalities and overcoming systemic barriers to efficacy. Our findings highlight the intricate interplay between endolysins and bacterial targets, underscoring the importance of further research to elucidate underlying mechanisms and refine therapeutic utility. Overall, our study provides valuable insights into the burgeoning field of endolysin-based antimicrobial therapies, offering promising avenues for the development of novel strategies to combat “ESKAPEE” infections.

## Data availability statement

The raw data supporting the conclusions of this article will be made available by the authors, without undue reservation.

## Ethics statement

The animal study was approved by the Institutional Animal Care and Use Committee at Nanjing University of Chinese Medicine. The study was conducted in accordance with the local legislation and institutional requirements.

## Author contributions

WC: Conceptualization, Investigation, Methodology, Writing – original draft, Writing – review & editing. L-MH: Investigation, Methodology, Writing – original draft, Writing – review & editing. X-ZC: Methodology, Validation, Writing – original draft, Writing – review & editing. P-CY: Methodology, Writing – original draft, Writing – review & editing. HL: Methodology, Writing – original draft, Writing – review & editing. Y-YR: Methodology, Writing – original draft, Writing – review & editing. J-HG: Methodology, Supervision, Writing – original draft, Writing – review & editing. C-YZ: Methodology, Visualization, Writing – original draft, Writing – review & editing. JH: Methodology, Resources, Writing – original draft, Writing – review & editing. W-XW: Data curation, Methodology, Writing – original draft, Writing – review & editing. Z-LH: Data curation, Formal analysis, Supervision, Validation, Writing – original draft, Writing – review & editing. C-MH: Funding acquisition, Project administration, Supervision, Writing – original draft, Writing – review & editing.

## Supplementary material

SUPPLEMENTARY FIGURE S1 CHU-1 exhibits synergistic interaction with polymyxin B against *A. baumannii in vitro*. Bacteria without any treatment served as a growth control.

SUPPLEMENTARY FIGURE S2 Effect of temperature, pH, salt concentration and metal ions on the lytic activity of CHU-1.
