# Supplementary material for: Engineered endolysin of Klebsiella pneumoniae phage is a potent and broad-spectrum bactericidal agent against “ESKAPEE” pathogens
Source: Front Microbiol. 2024 May 9;15:1397830. doi: 10.3389/fmicb.2024.1397830 (PMC11112412; doi:10.3389/fmicb.2024.1397830)
Supplement: Supplementary file 3 [file Table_3.docx]

Table S3. MIC and MBC of Zinc ion against six “ESKAPE” strains.

| Strains | MIC (mM) | MBC (mM) |
| --- | --- | --- |
| *Acinetobacter baumannii* YQ4 | 2 | >16 |
| *Enterobacter cloacae* 7-26 | 1.5 | 3 |
| *Enterococcus faecalis* 10-17 | >16 | >16 |
| *Klebsiella pneumoniae* 84 | 2 | 4 |
| *Psedomonas aeruginosa* 1-22 | 4 | >16 |
| *Staphylococcus aureus* 14-32 | 3 | 24 |
